# Supplementary material for: Effect of Size and Shape on Electrochemical Performance of Nano-Silicon-Based Lithium Battery
Source: Nanomaterials (Basel). 2021 Jan 25;11(2):307. doi: 10.3390/nano11020307 (PMC7912472; doi:10.3390/nano11020307)
Supplement: Supplementary file 1 [file nanomaterials-11-00307-s001.pdf]

Supplementary Information

Effect of Size and Shape on Electrochemical Performance of Nano-Silicon-Based Lithium Battery

Caroline Keller [1,2], Antoine Desrues [3], Saravanan Karuppiah [1,2], Eléa Martin [1], John P. Alper [2,3], Florent Boismain [3], Claire Villevieille [1], Nathalie Herlin-Boime [3], Cédric Haon [2] and Pascale Chenevier [1,*]

[1] University Grenoble Alpes, CEA, CNRS, IRIG, SYMMES, STEP, 38000 Grenoble, France; caroline.keller@univ-grenoble-alpes.fr (C.K.); krsaro87@gmail.com (S.K.); elea.martin@etu.unistra.fr (E.M.); claire.villevieille@cea.fr (C.V.)

[2] University Grenoble Alpes, CEA, LITEN, DEHT, 38000 Grenoble, France; johnalper@googlemail.com (J.P.A.); cedric.haon@cea.fr (C.H.)

[3] University Paris Saclay, CEA, CNRS, IRAMIS, NIMBE, LEDNA, 91191 Gif-sur-Yvette, France; antoine.desrues@cea.fr (A.D.); florent.boismain@insa-lyon.fr (F.B.); nathalie.herlin@cea.fr (N.H.-B.)

* Correspondence: pascale.chenevier@cea.fr

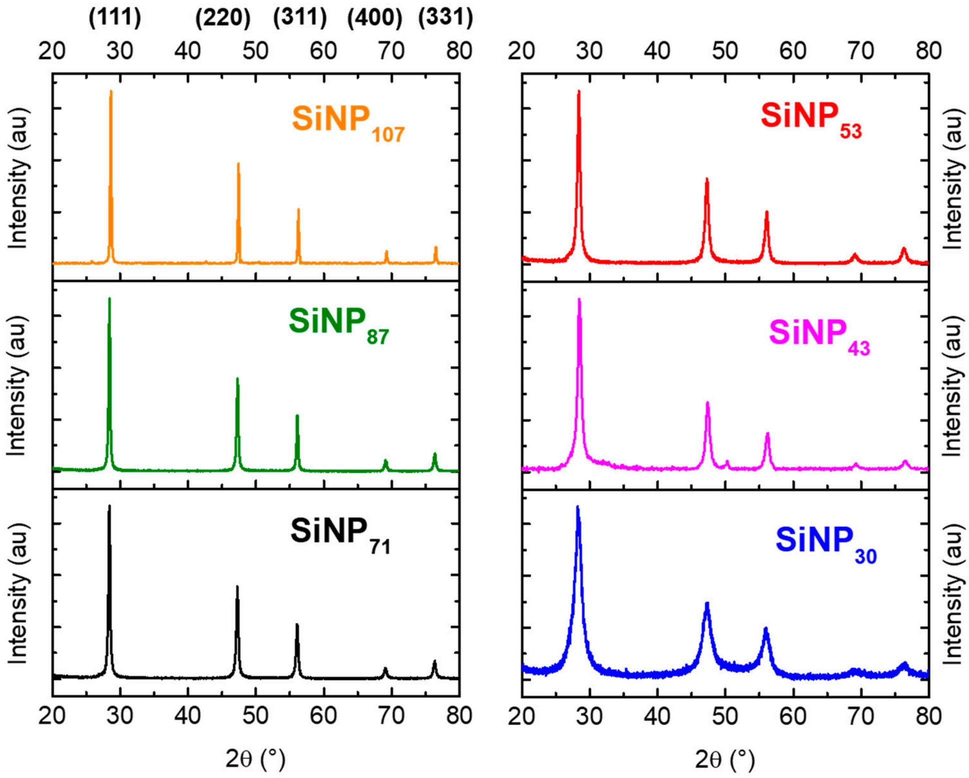

Figure 1. X-ray diffractograms of the SiNP samples.

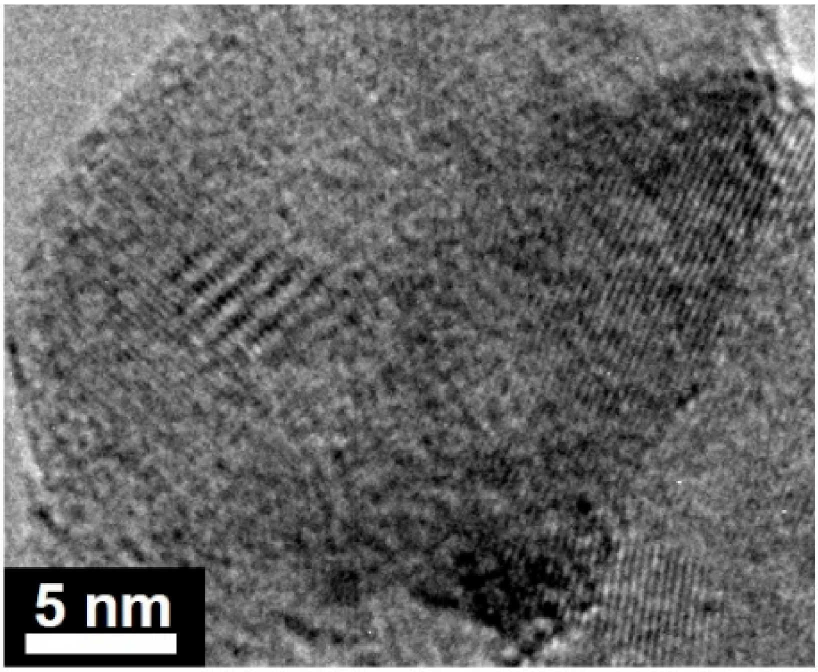

Figure 2. HRTEM images of $SiNP_{30}$.

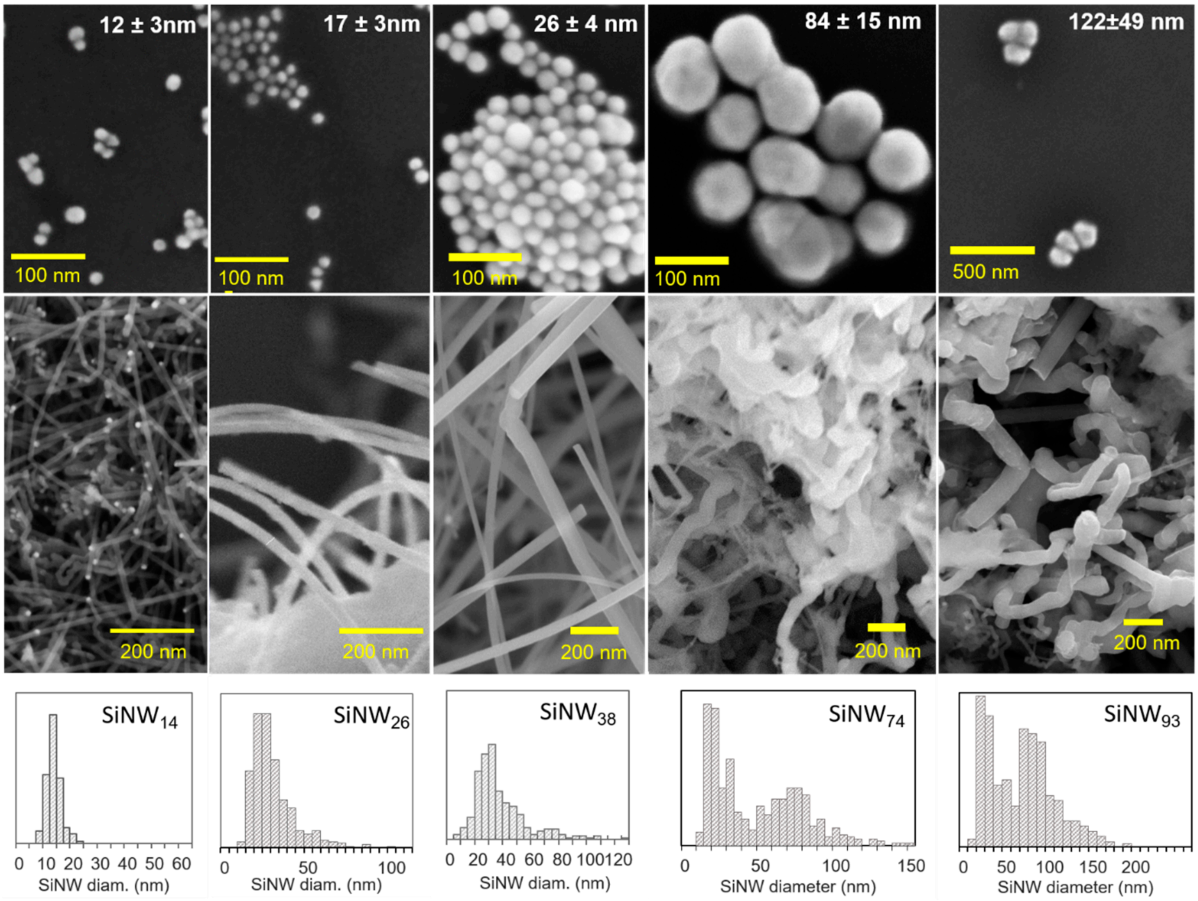

Figure 3. SEM images of the AuNP catalysts and of the SiNWs grown subsequently obtained, corresponding histograms of the SiNW diameter.

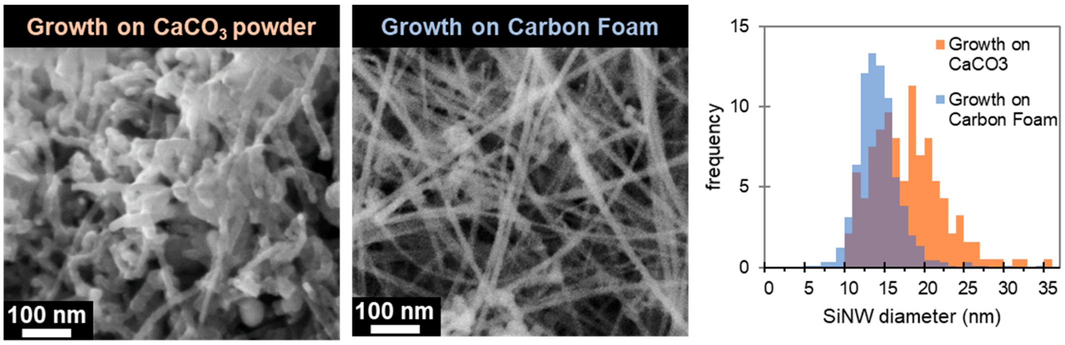

Figure 4. SEM images and corresponding diameter histogram of SiNWs grown from 12nm AuNPs on $CaCO_3$ nanopowder or in a $1cm^3$ carbon foam cube.

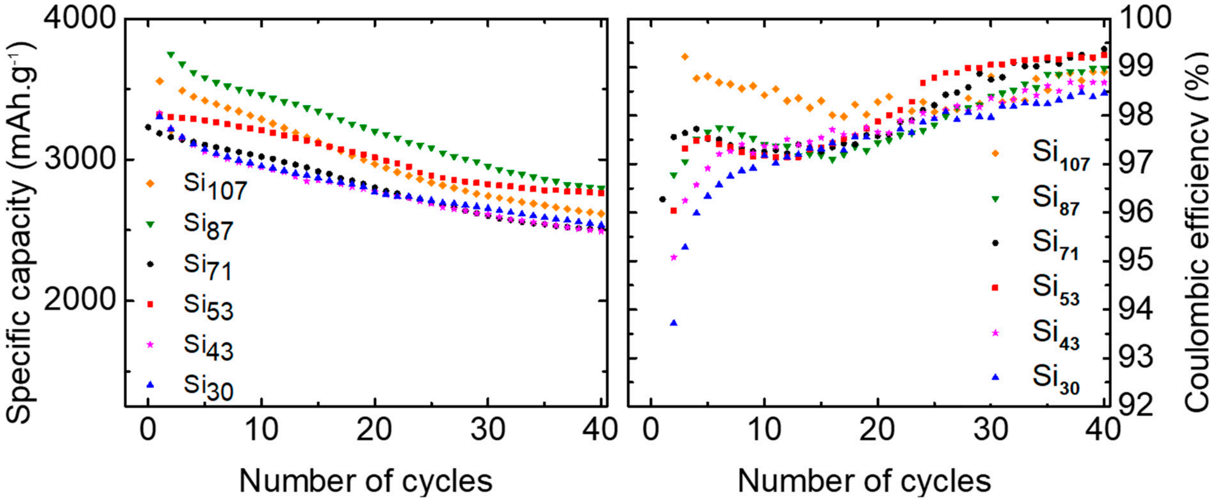

Figure S5. Specific capacity and coulombic efficiency for all SiNPs presented in Table 1.

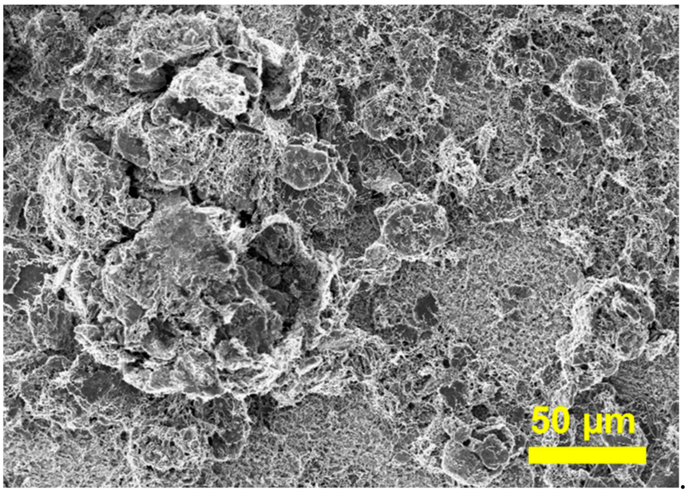

Figure 6. SEM image of an electrode with $SiNW_9$.

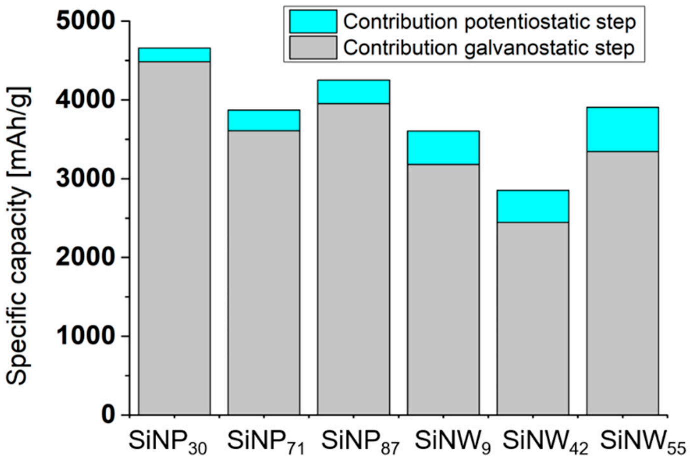

Figure 7. Specific capacity obtained in the first lithiation during the galvanostatic (grey) and the potentiostatic (light blue) steps for SiNPs and SiNWs.

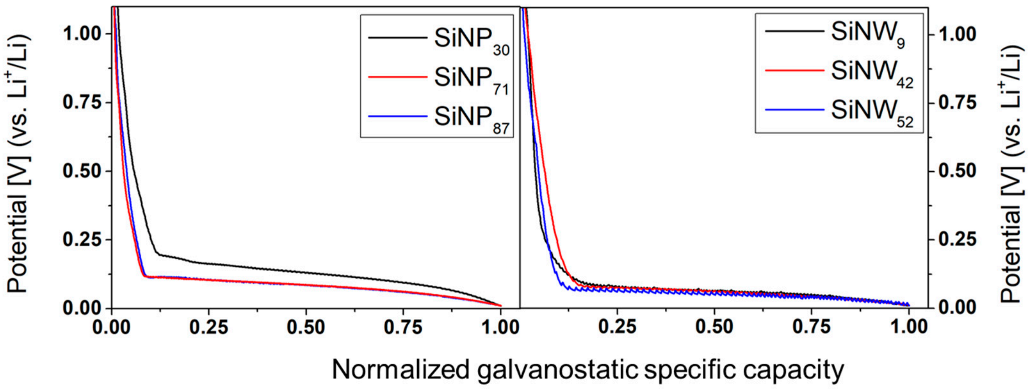

Figure 8. Cell potential vs Li^+/Li as a function of normalized galvanostatic capacity for SiNPs (left) and SiNWs (right) at first cycle.
